# Structure, function and assembly of nuclear pore complexes

**DOI:** 10.1038/s41580-025-00881-w

**Published:** 2025-09-09

**Authors:** Stefan Petrovic, George W. Mobbs, André Hoelz

**Affiliations:** 1 California Institute of Technology, Division of Chemistry and Chemical Engineering, 1200 East California Boulevard, Pasadena, CA 91125, USA; 2 Howard Hughes Medical Institute, California Institute of Technology, 1200 East California Boulevard, Pasadena, CA 91125, USA

## Abstract

The defining property of eukaryotic cells is the storage of heritable genetic material in a nuclear compartment. For eukaryotic cells to carry out the myriad biochemical processes necessary for their function, macromolecules must be efficiently exchanged between the nucleus and cytoplasm. The nuclear pore complex (NPC) — which is a massive assembly of ~35 different proteins present in multiple copies totaling ~1,000 protein subunits, and architecturally conserved across eukaryotes — establishes a size-selective channel for regulated bidirectional transport of folded macromolecules and macromolecular assemblies across the nuclear envelope. In this Review, we present an overview of the insights gained from recent near-atomic composite NPC structures, and their significance towards understanding the functions of the NPC. We discuss advances in our understanding of the permeability barrier, modes of nucleocytoplasmic transport, and the mobile transport factors involved. Finally, we present future research directions aimed at elucidating the nuclear basket architecture, mechanisms of mRNA export, NPC biogenesis, and mechanosensation.

## Introduction

The nuclear pore complex (NPC) is a eukaryotic cellular structure that regulates bidirectional traffic of macromolecules across the nuclear envelope. Solving the intricate structure of this ~120MDa protein complex in humans, and understanding how it mediates export of mature mRNA, regulates signal transduction into the nucleus, and contributes to the biogenesis of the replication, transcription, and splicing machineries, is essential to understand the eukaryotic central dogma.

The concept of nucleocytoplasmic transport emerged in the mid-20^th^ century, when electron microscopy (EM) observations of nuclear pores indicated that NPCs function as macromolecular gatekeepers^1–4^. These observations were followed by three phases of research and discoveries: (*1*) EM-based observation of NPCs; (*2*) molecular characterization of the NPC and nucleocytoplasmic transport machinery components; and (*3*) integrative structural biology leading to near-atomic structures of entire NPCs.

In this Review, we discuss how biochemical reconstitution, structural biology and cell biology have converged to determine and validate near-atomic composite structures of intact NPCs. We describe recent structural and biochemical detail that are providing a deeper understanding of the protein-protein interactions that enable the NPC architecture to be remarkably dynamic, and the implications for the selective transport of macromolecules in and out of the nucleus, as well as for physiological functions such as the establishment of a size selective permeability barrier and mechanosensation. Lastly, we address the remaining challenges in characterizing poorly understood structural elements of the NPC, as well as mechanisms of mRNA export and NPC biogenesis and outline open questions that will guide future research.

### The structure of the NPC

**Figure 1 |:**
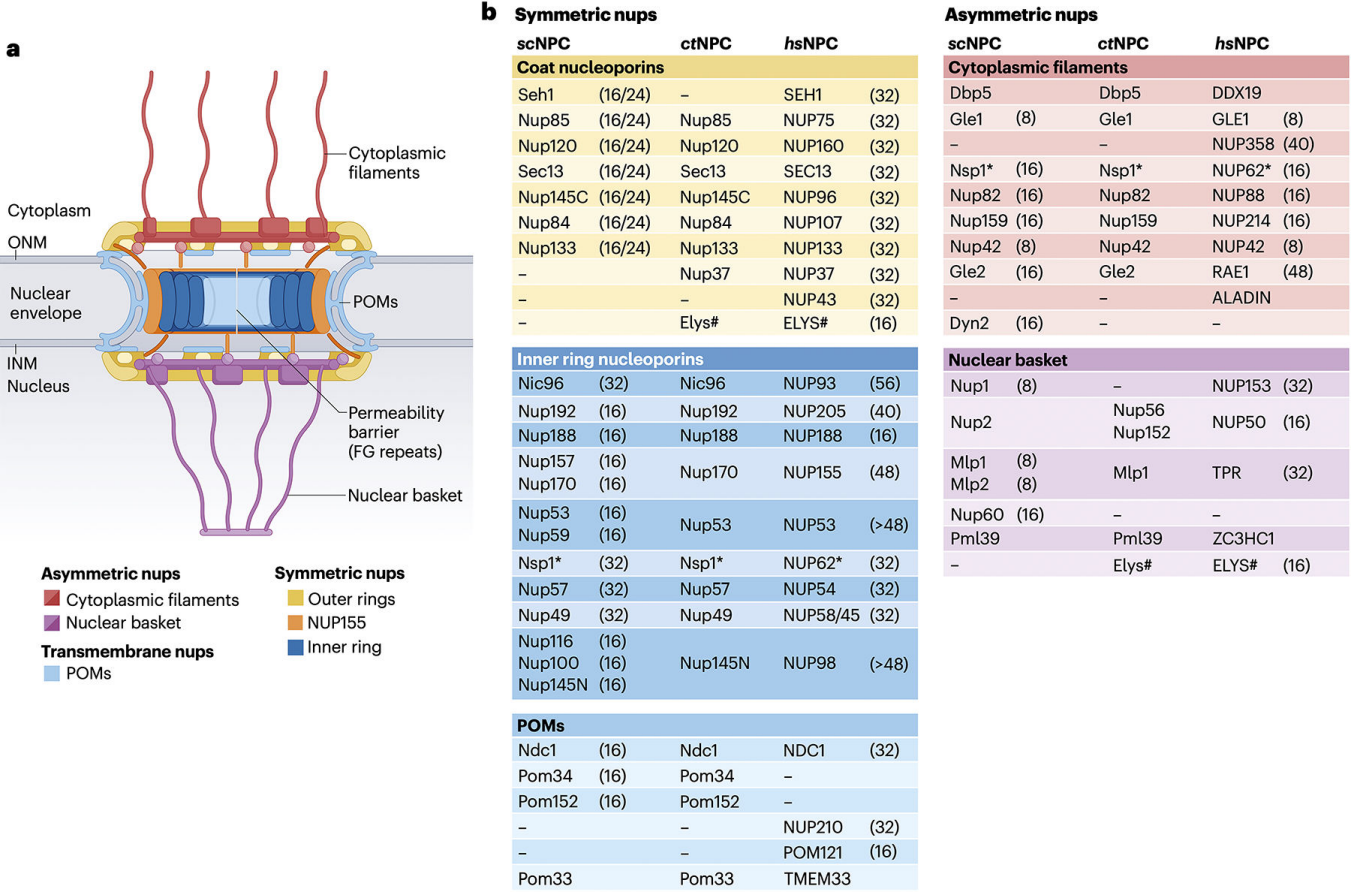
Overview of NPC architecture. **a** | Schematic representation of NPC architecture. A cutaway view depicting half of an NPC is shown. **b** | Nomenclature for nups in *Saccharomyces cerevisiae* (*sc*), *Chaetomium thermophilum* (*ct*), and *Homo sapiens* (*hs*) NPCs, grouped by subcomplex. Asterisks (∗) indicate that NUP62 and its homologs are also found in the cytoplasmic filaments. Hash (#) indicates that ELYS is exclusively a member of the nuclear outer ring coat nup complex (CNC) in humans, rather than being symmetrically distributed. However, its distribution in other species may vary, as *Schizosaccharomyces pombe* homolog is observed in both nuclear and cytoplasmic outer rings. Established nup stoichiometries in intact NPCs are indicated in parentheses. In *S. cerevisiae,* CNC component stoichiometries vary depending on the presence of a single or double nuclear outer ring^21,28,112,114^. Abbreviations: NPC, nuclear pore complex; POMs, nups of the pore membrane domain; ONM, outer nuclear membrane; INM, inner nuclear membrane.

The NPC is one of the largest supramolecular assemblies in eukaryotic cells, with a mass ranging from ~60MDa and ~500 proteins in fungi, to ~120MDa and ~1,000 proteins in vertebrates^5–9^. Early EM studies revealed an 8- and 2-fold rotationally “symmetric core” along the nucleocytoplasmic axis and across the nuclear envelope plane, respectively^10–12^. Nuclear pores are circular openings in the nuclear envelope created by fusion of the outer (ONM) and inner (INM) nuclear membranes, generating cylindrical channels across the nuclear envelope that are lined by the NPC’s inner ring within the pore channel and framed by two outer rings resting atop the INM and ONM surfaces; collectively, the inner and outer rings constitute the NPC’s symmetric core (Fig. 1A). Transmembrane nucleoporins (nups) in the pore membranes termed integral “pore membrane domain” proteins (POMs) present both nuclear pore-facing and nuclear envelope lumen-facing soluble domains. Asymmetric cytoplasmic filament and the nuclear basket structures adjoin the symmetric core. This architecture comprises ~35 evolutionarily conserved nups (Fig. 1B)^13,14^. Following convention, human nup names are reported in uppercase (e.g., NUP205), whereas homologs from other organisms use sentence case (e.g., Nup192).

#### Evolutionary conservation of the NPC architecture

**Figure 2 |:**
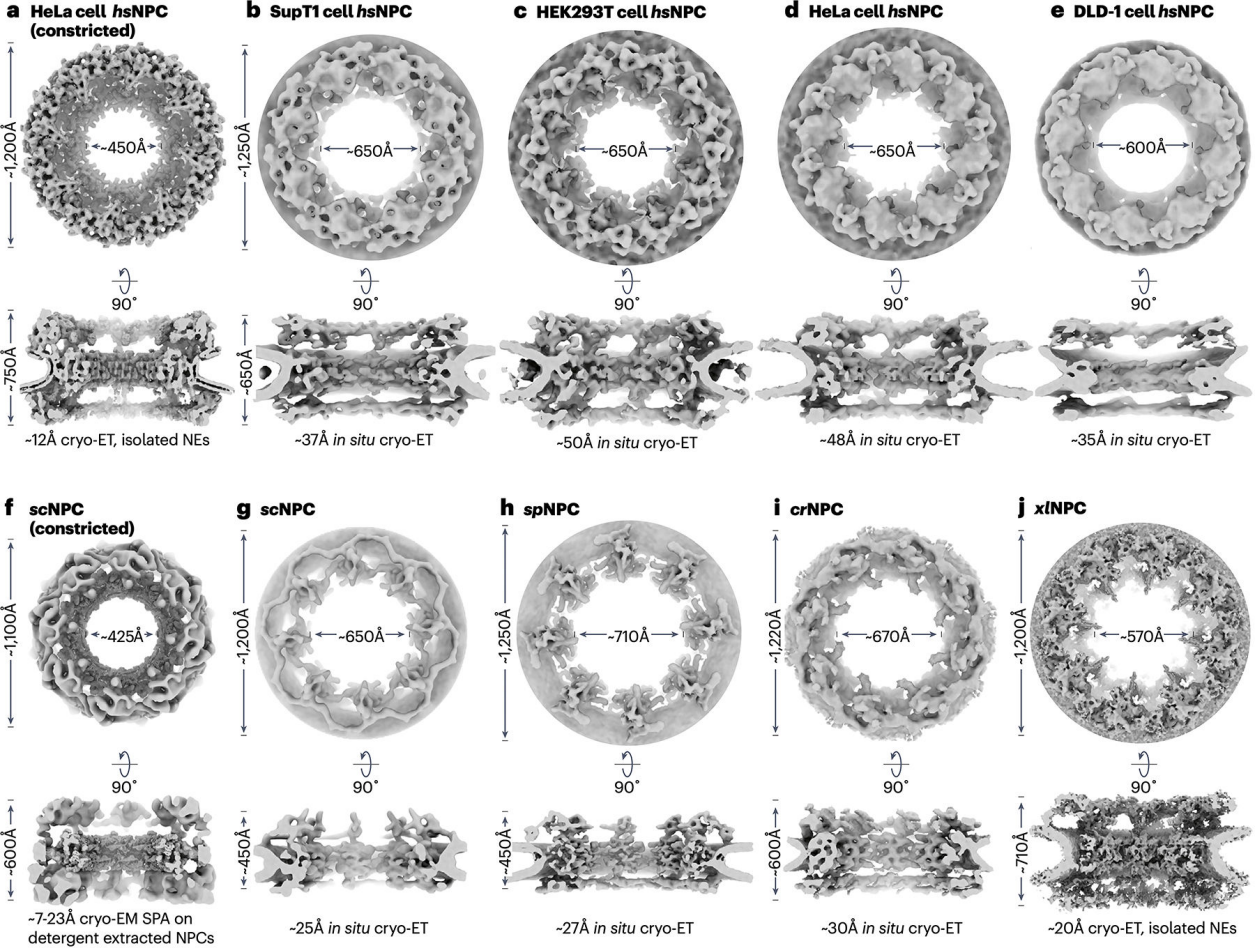
Comparison of cryo-EM/ET NPC reconstructions. Scaled isosurface representations of averaged cryo-EM/ET reconstructions of NPCs from various species determined either *in situ* from cryo-FIB milled specimens, upon purification in the presence of detergents, or in the context of an isolated nuclear envelope (NE). NPC reconstructions display the cytoplasmic outer rings, and are rotated 90^o^ to a cutaway view in the plane of the NE. **a** | HeLa cervical cancer cell *hs*NPC (constricted) from isolated NEs (EMD – 14322)^24^. **b** | SupT1 T-cell lymphoma *in situ hs*NPC **(**EMD – 11967)^32^. **c** | HEK293 embryonal kidney cell *in situ hs*NPC (EMD – 14321)^24^. **d** | HeLa cervical cancer cell *in situ hs*NPC (EMD – 14327)^24^. **e** | DLD-1 colorectal adenocarcinoma cell *in situ hs*NPC (EMD – 12814)^25^. **f** | Detergent-extracted *sc*NPC (EMDs – 41114, 41116, 41117, 41119, and 41300)^29^. **g** | *In situ sc*NPC (EMD – 10198)^22^. **h** | *In situ sp*NPC (EMD – 11373)^23^. **i** | *In situ cr*NPC (EMD – 4355)^20^. **i** | *xl*NPC from isolated NEs (EMDs - 3005, 3006, 3007, 3008, and 3009)^17^. Abbreviations: cryo-EM, cryo-electron microscopy; cryo-ET, cryo-electron tomography; cryo-FIB, cryo-focused ion beam; EMD, Electron Microscopy Databank; NE, nuclear envelope; NPC, nuclear pore complex; *hs*, *Homo sapiens*; *sc*, *Saccharomyces cerevisiae; sp*, *Schizosaccharomyces pombe*; *cr*, *Chlamydomonas reinhardtii*; *xl*, *Xenopus laevis*.

Despite a conserved general architecture, NPC dimensions and composition vary between species. Cryogenic electron microscopic or tomographic (cryo-EM/ET) reconstructions of intact NPCs, either isolated or *in situ* from cryogenic focused ion beam (FIB)-milled specimens, from *Homo sapiens* (*hs*), the frog *Xenopus laevis* (*xl*), the unicellular fungi *Saccharomyces cerevisiae* (*sc*) and *Schizosaccharomyces pombe* (*sp*), the unicellular alga *Chlamydomonas reinhardtii* (*cr*), and the amoeba *Dictyostelium discoideum* (*dd*) have been determined at resolutions that permit interpretation of 3D features by comparison with experimental or predicted atomic models of individual nups and nup complexes (Fig. 2)^15–31^. Moreover, cryo-ET maps of *hs*NPCs from macrophages, and HeLa, HEK293, DLD-1, and SupT1-R5 cancer cell lines, highlight the general invariance of the *hs*NPC architecture^24,32,33^. Although early studies of nup interactions primarily used *H. sapiens* and *S. cerevisiae* nups, the nups of the thermophilic fungus *Chaetomium thermophilum* (*ct*) enabled the reconstitution of intact *ct*NPC subcomplexes and their underlying nup-nup interaction network, which subsequent research has shown to be evolutionarily conserved^19,26,27,34–42^. Nevertheless, a subtomogram averaged *ct*NPC cryo-ET map has not yet been achieved^19,26,27,34–42^.

The availability of these data allows us to compare NPCs between and within species. Whereas the layered inner ring architecture is evolutionarily invariant, the number of concentric rings formed by the head-to-tail arrangement of “Y”-shaped outer ring protomers called coat nup complexes (CNCs), varies. Vertebrate *hs*/*xl*NPCs have double rings on both cytoplasmic and nuclear sides^15,17,43^. Whereas, the *dd*NPC has a single cytoplasmic outer ring, and triple nuclear outer ring arrangement^31^. The *cr*NPC and *sp*NPC have a single cytoplasmic and double nuclear outer ring, except that the *sp*NPC’s cytoplasmic outer ring is discontinuous lacking the “Y” stalks^23^. The *sc*NPC has a single cytoplasmic outer ring and either single or, less commonly, double nuclear outer rings^22,28–30^. Though structurally uncharacterized, even evolutionarily distant protist excavates and alveolates possess a conserved set of symmetric core nups, suggesting a common NPC origin^44–46^.

#### The symmetric core

**Figure 3 |:**
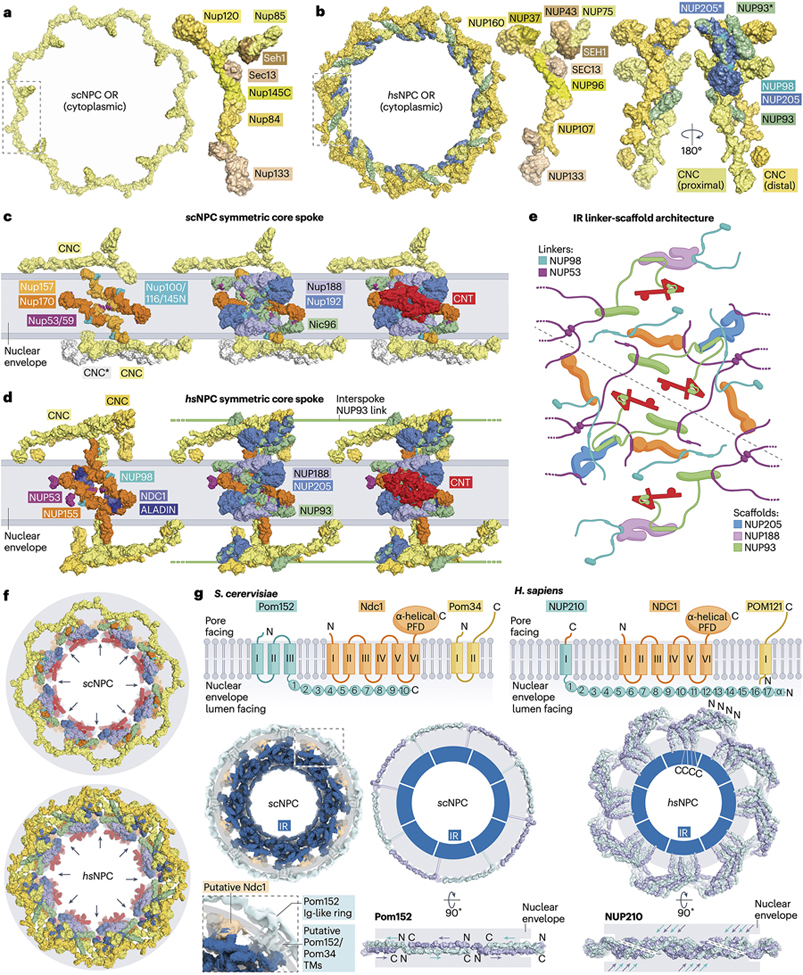
The NPC symmetric core. Surface representation of cytoplasmic outer ring (OR) views with, close-up views of the underlying protomers (inset box) in **a** | the *sc*NPC, and **b** | the *hs*NPC, where additional NUP205 and NUP93 molecules indicated by an asterisk (*) are present in the outer rings^27^. Surface representation of NPC symmetric core spokes, highlighting the layered inner ring (IR) architecture from the NE (left) to the central transport channel (right) in **c** | *S. cerevisiae*, where a second CNC ring indicated by the asterisk (*) is present in the nulcear OR of some NPCs, and **d** | *H. sapiens*^27^. **e** | Cartoon schematic showing the inner ring’s linker-scaffold architecture, colored as in panel **d**. Linker binding sites on scaffold nups are denoted by circles and joined by lines representing linker nups; 2-fold symmetry axis indicated in grey. Figure adapted from Petrovic *et al*^27^. **f** | Cytoplasmic view of dilated the *sc*NPC and *hs*NPC with lateral channels between the symmetric spoke of the inner ring that can accommodate the passage of membrane proteins indicated with arrows^27^. **g** | Cartoon schematic of the domain organization and membrane topology of the *S. cerevisiae* and *H. sapiens* POMs, along with cutaway isosurface representations of cryo-EM/ET reconstructions of the inner ring and POM regions from *sc*NPC (EMDs – 41114, 41116, 41117, 41119, and 41300)^29^ and surface representations of low-resolution modeling of the ring structures formed by Ig-like domains of Pom152 and NUP210, respectively. Abbreviations: NE, nuclear envelope; NPC, nuclear pore complex; *hs*, *Homo sapiens*; *sc*, *Saccharomyces cerevisiae; xl*, *Xenopus laevis;* OR, outer ring; IR, inner ring; PFD, pore facing domain; TMs, transmembrane α-helices.

Although the CNC’s composition and shape were determined by biochemical isolation from endogenous sources,^47^ its architectural role in the NPC was understood by matching the Y-shaped crystal structure of a reconstituted *S. cerevisiae* CNC to shapes in the outer rings of a ~32Å *hs*NPC cryo-ET map^15,43^. The intact ~580kDa *sc*CNC comprises seven nups: the two arms consisting of Nup120 and Nup85•Seh1 (macromolecular complex interactions are denoted with •), and the stem consisting of Nup145C•Sec13•Nup84•Nup133, connected at a triskelion-like junction (Fig. 3A)^43,48–57^. The human CNC “Y” architecture is conserved but has additional nups decorating the arms (Fig. 3B)^16,18,19,24,58–60^. CNC interactions with the nuclear envelope are mediated by amphipathic lipid packing sensor (ALPS) motifs in Nup120/NUP160 and Nup133/NUP133^61^. In *S. cerevisiae*, the head-to-tail arrangement necessary to form continuous outer rings is mediated by an unstructured N-terminal linker region of Nup133 that had been shown to bind Nup120, with the *in vivo* disruption of this interaction causing a NPC clustering phenotype typical of incompletely assembled NPCs^54^. The homologous *C. thermophilum* Nup133 region is necessary for the liquid-liquid phase separation (LLPS) of CNCs reconstituted at physiological concentrations^19,26^.

A significant breakthrough in understanding the symmetric core architecture came from docking additional crystal structures and derived homology models into improved cryo-ET maps, concurrent with biochemical mapping and physiological validation of inner ring nup-nup interactions^18,19,34,36,37,43,62^. Inner ring nups include folded scaffolds (Nup192/NUP205, Nup188/NUP188, Nup170/NUP155, Nic96/NUP93’s C-terminal domain, Nsp1/NUP62, Nup49/NUP58, and Nup57/NUP54) and mostly unstructured linkers that contain multiple scaffold-binding sites (Nup145N/NUP98, Nup53/NUP53, and Nic96/NUP93’s N-terminal region). Two alternative hetero-octameric inner ring complexes (IRCs) could either be reconstituted from recombinant *ct-*nups or isolated from *Xenopus* oocyte egg extract, centered around the question mark-shaped Nup192/NUP205 and Nup188/NUP188 hubs^19,34,37,38,62^. Inner rings of all known NPCs can be thought of as concentric scaffold cylinders connected by linkers that arise from sixteen copies of each Nup192/NUP205- and Nup188/NUP188-IRCs arranged into eight spokes containing two copies of each IRC related by a two-fold symmetry operator (Fig. 3C,D)^18,19^. In the *hs*NPC, additional NUP155 are found bridging the inner ring and outer rings^16,19^.

The concept of linker nups sustaining the NPC’s architecture was first formulated in the context of the Nup120-Nup133 interaction in the outer ring^54^, and was subsequently expanded to include the Nup159-Nup82 interaction in the cytoplasmic filaments^63^, as well as the cohesion of the nucleoporins in the inner ring^19,34,36–38^. A salient inner ring feature is Nic96’s unstructured N-terminal region, which recognizes the stoichiometry and fold of the “4”-shaped Nsp1•Nup49•Nup57 channel nup trimer (CNT) coiled-coil complex, positioning it to project its unstructured phenylalanine-glycine (FG) repeat regions from the equator of the central transport channel^18,19,27,37,38,62,64^. Distance restraints obtained through systematic biochemical mapping and by docking experimental scaffold•linker structures into cryo-ET maps of *hs*/*sc*NPCs allowed for the entire linker-scaffold network to be traced^27^. A parallel approach employing artificial intelligence (AI)-based modeling identified the majority of linker-scaffold interactions, producing a nearly equivalent model^24^. Linker-like densities were subsequently observed in improved cryo-EM maps of detergent-extracted isolated *sc*NPCs, consistent with the established linker-scaffold network^28,29^.

Composite *hs*/*sc*NPC structures obtained by docking near-atomic structures into cryo-EM/ET maps of NPCs imaged from isolated nuclear envelopes and detergent extracted preparations enabled the interpretation of lower resolution *in situ* cryo-ET maps of the *hs*/*sc*NPCs whose central transport channel is dilated (Fig. 2)^22,24,25,27,28,32^. Most linker-scaffold interactions form between nups of the same symmetric spoke subunit, except for Nup53/NUP53’s long flexible linkers which present a homodimerizing interface between spokes (Fig. 3E). This arrangement allows the inner ring to dilate by up to ~200Å in response to tension in the nuclear envelope, with the eight spokes moving as rigid bodies to create gaps between them.

Beyond resolving the long-standing riddle about how the NPC’s central transport channel may exhibit varying diameters, this observation pinpoints the location of lateral channels that permit the transit of integral membrane protein cargos between the ONM and the INM (Fig. 3F)^5,12,23,24,27,65–68^. In contrast, outer rings are more rigidly organized, with scaffold-scaffold interactions minimizing movement between dilated and constricted states. Additional copies of NUP205 and NUP93 are found in the outer rings of *hs*/*xl*NPCs^24,27,69,70^, forming trans-spoke staples that reinforce the head-to-tail arrangement of outer ring protomers (Fig. 3B & D), breaking the two-fold symmetry of symmetric core spokes through the absence of a second NUP205-NUP93 copy in the nuclear outer ring. Similarly, orphan densities present at double nuclear outer rings of the *sc*NPC have been attributed to *sc*Nic96^29^. The linker-scaffold architectural principle allows for many interactions to be mediated by few flexible entities, conferring not only mechanical plasticity where necessary but also a way to rapidly disassemble a large protein assembly through the phosphorylation of select linear motifs (see the Mitotic NPC disassembly section below)^71–74^.

#### The NPC’s transmembrane portion

In addition to soluble nups, the NPC recruits a few integral membrane proteins – the POMs (Fig. 3G). The only essential POM, Ndc1/NDC1, is conserved from fungi to humans, whereas Pom152 and Pom34 are architecturally similar but not directly homologous to NUP210 and POM121. POMs consist of transmembrane α-helices and soluble regions facing either the nuclear envelope’s lumen (NUP210 and Pom152) or the pore (NDC1/Ndc1, POM121, and Pom34)^5,7^.

Ndc1/NDC1 has six transmembrane α-helices and a pore-facing soluble domain that are modeled to exist in a stoichiometry of two copies per spoke according to low-resolution cryo-EM/ET density of *hs*/*sc*NPC inner rings^24,28,75^. Pulldowns from detergent-solubilized lysates identified Ndc1•Pom34•Pom152 and Ndc1•Nup53/59 complexes^76,77^. Docking of AI-predicted structures into low-resolution cryo-EM/ET density, supported by chemical crosslinking restraints, places the pore-facing domain of NDC1 in proximity of ALADIN, NUP155, and NUP53 in the inner ring (Fig. 3D,G)^24,78^.

NUP210 and Pom152 both project immunoglobulin (Ig)-like domains into the lumen generated by the double membrane of the nuclear envelope^79^. They differ, however, in the number of Ig-like domains, the number of transmembrane α-helices, and in their domain order. NUP210’s single transmembrane α-helix and a C-terminal amphipathic α-helix are sufficient for NPC recruitment, though their binding partners are unknown (Fig. 3G)^80^. Cryo-EM/ET maps reveal spatial differences in the arrangement of the Ig-like domains: two copies of Pom152 per spoke pair in an antiparallel fashion to form a ring in the *sc*NPC, whereas eight copies of NUP210 per spoke form a butterfly-like arrangement in the *hs*/*xl*NPC (Fig. 3G)^21,24,28,29,81^.

POM121 and Pom34, though not homologous, are respectively single- and double-pass transmembrane proteins with large pore-facing unstructured regions^82,83^. POM121 has been shown to bind to NUP155 and the *hs*CNC *in vitro*^15,84^. The locations of POM121 and Pom34 remain unverified. However, the Pom34 and Pom152 transmembrane α-helices are predicted to form a 2:2 heterotetramer corresponding to tubular cryo-EM density in the nuclear envelope at the inner ring’s equator^29^. A fourth potential POM, Pom33, was missed by mass-spectrometry-based NPC inventories and is not identifiable in current *sc*NPC cryo-EM/ET maps, yet it colocalizes with clustered NPCs in *nup133*Δ *S. cerevisiae* strains^13,85^. Pom33 and its presumed homolog TMEM33 are predicted to be multipass integral membrane proteins with short unstructured N- and C-terminal pore-facing regions^85,86^.

Despite some POM-nup interactions having been characterized or modeled in low-resolution cryoEM-/ET maps by AI prediction, systematic and *in vivo* validated mapping of the POM-nup interactome is lacking, and interactions mediating the NPC’s nuclear envelope-anchoring remain to be discovered.

#### The cytoplasmic face

**Figure 4 |:**
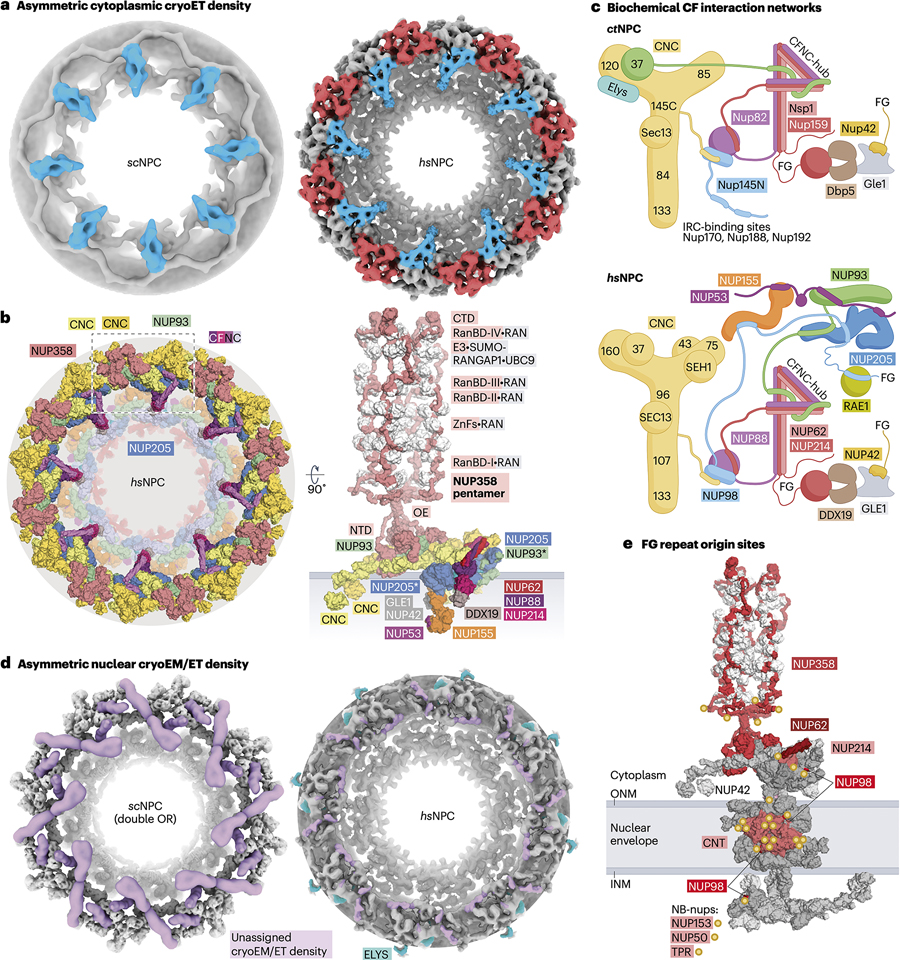
The asymmetric portions of the NPC. **a** | Isosurface representations of cryo-EM/ET reconstructions of the cytoplasmic face of the *sc*NPC (EMDs – 10198)^22^ and *hs*NPC (EMD – 14322)^24^. Asymmetric density clusters correspond to the CFNC (blue) and NUP358 (red)^24,29^. **b** | Surface representation of the cytoplasmic face of the *hs*NPC, close-up view of an isolated cytoplasmic face protomer illustrating the attachment of the CFNC, and the pentameric NUP358 bundles held together by interactions between homotypic oligomerization elements (OEs)^26^. **c** | Schematic representation summarizing nup-nup interaction networks characterized by biochemical reconstitution with purified recombinant *C. thermophilum* and *H. sapiens* nups, illustrating CFNC architecture and its attachment to the OR^26^. **d** | Isosurface representations of cryo-EM/ET reconstructions of the nuclear face of the *sc*NPC (EMDs – 41114, 41116, 41117, 41119, and 41300)^29^ and *hs*NPC (EMD – 14322)^24^. Structurally uncharacterized regions corresponding to ELYS (cyan) and unassigned proteins (purple) are highlighted^24,29^. **e** | Origin points for FG-repeat regions denoted by yellow circles are idicated on a surface representation of a single *hs*NPC spoke encompassing the inner ring, outer rings, and cytoplasmic filaments^26^. Abbreviations: NPC, nuclear pore complex; *hs*, *Homo sapiens*; *sc*, *Saccharomyces cerevisiae; ct*, *Chaetomium thermophilum;* OR, outer ring; CNC, coat nup complex; CFNC, cytoplasmic filament nup complex; CTD, C-terminal domain; NTD, N-terminal domain; RanBD, Ran binding domain; ZnF, Zinc finger domain; OE, oligomerization element; NB-nups, nuclear basket nups.

Asymmetrically distributed cytoplasmic filament nups are crucial for nucleocytoplasmic transport. Recent cryo-EM/ET studies have increased the resolution of the *hs*/*xl*NPCs’ cytoplasmic face, allowing for its interpretation using high-resolution experimental structures, biochemical reconstitution, AI-based structure prediction, and *in vivo* validation^24,26,69,70^. Maintaining the eight-fold symmetry of the outer rings they are attached to, eight instances of two spatially segregated protein clusters decorate the cytoplasmic face of *hs*/*xl*NPCs (Fig. 4A).

The first cluster corresponds to a NUP358 homopentamer, four of whose α-helical solenoid N-terminal domains interact with the “stalk” portions of two tandem-arranged CNCs in the outer ring. The fifth copy sits atop breaking the two-fold symmetry. Within this dome-like structure resides NUP93’s α-helical solenoid (Fig. 4B)^24,26,69,70^. The NUP358 homopentamer is held together by a homotypic coiled-coil oligomerization element required for NPC localization^26,69^. NUP358’s >3,000 residues form a flexible array of domains projecting ~60nm into the cytoplasm to generate a forest of 480 RAN-binding and zinc finger (ZnF) domains that recruit RAN^26,87,88^, while also attaching the RAN-GTPase activating protein 1 (RANGAP1) that facilitates disassembly of export and prevents premature disassembly of import complexes (see The permeability barrier of the NPC section below)^89,90^.

The second cluster forms from the evolutionarily conserved modular architecture of the hetero-octameric cytoplasmic filament nup complex (CFNC). The modules are biochemically tractable: a subcomplex formed by Nup159/NUP214, Gle1/GLE1, Nup42/NUP42, and the DEAD-box helicase Dbp5/DDX19 on one side, and a subcomplex formed by Nup82/NUP88, Nup159/NUP214, Nup145N/NUP98, and Gle2/RAE1 on the other (Fig. 4C)^24,26,40,41,63,64,91–104^. RNA-binding has been mapped onto both modules^26,41,93,99,100^. The two modules are held together by a trimeric coiled coil of Nsp1/NUP62, Nup82/NUP88, and Nup159/NUP214 (CFNC-hub), whose three-stalked architecture reminiscent of the inner ring’s CNT is apparent in cryo-EM/ET maps^24,26,69,70^. Functional implications of this arrangement in mRNA export are discussed in the mRNA export section. How the CFNC is attached to the cytoplasmic outer ring diverges across species. In the *hs*NPC, NUP93 recruits the CFNC-hub analogously to the CNT in the inner ring^26^. Based on the presence of two NUP93 copies per cytoplasmic outer ring spoke of the *hs*/*xl*NPC, two CFNC copies per spoke would be expected. However, cryo-EM/ET maps reveal the presence of a single well-resolved CFNC density, with some *xl*NPC cytoplasmic face reconstructions showing partially resolved density indicative of a second copy, possibly due to inherent flexibility or compositional heterogeneity^69,70,105^. The *in situ hs*NPC map reveals a potential second CFNC copy as well, or at least additional cryo-ET density of the appropriate size that nevertheless lacks detail for unambiguous assignment, albeit at a different location on the cytoplasmic face^24,26,32^. As in the inner ring, NUP205 is a linker-scaffold organizing hub at the cytoplasmic outer ring, coordinating the attachment of the CFNC through interactions with NUP98, NUP93, and NUP53 linkers (Fig. 4C). Biochemical reconstitution and mapping with *ct-*nups points to unstructured regions of its CNC components Nup37 and Nup145C acting as coiled-coil assembly sensors^26,40^. In the *sc*NPC, the CFNC architecture deviates by having multiple dynein light chain Dyn2 domains assist the CFNC-hubs in homodimerizing into characteristic “P”-shaped structures^22,103,104,106^. A high-resolution structure capturing the molecular details of a CFNC from any species with residue-level confidence has not yet been obtained.

#### The nuclear basket

At the NPC’s nuclear face, ~60–120nm basket-like structures have been observed by EM, yet continue to evade atomistic structural determination likely due to their inherent flexibility and compositional heterogeneity^107–110^. Similarly, efforts to ascertain the stoichiometry of nuclear basket nups have been inconsistent^21,111–113^. The nuclear basket has been implicated in excluding chromatin from the NPC’s periphery, selecting export-ready mRNAs and pre-ribosomal particles, protein quality control, and gene tethering^114–121^.

Rod-like densities near the nuclear outer ring in cryo-EM/ET maps of intact *hs*/*sc*NPCs have been proposed to correspond to nuclear basket attachment sites (Fig. 4D)^22,24,28^. Targeted subtomogram averaging of *in situ* cryo-ET reconstructions from *S. cerevisiae*, *Mus musculus*, *Toxoplasma gondii*, and *D. discoideum* have recently revealed eight filamentous structures protruding from the nuclear outer ring and additional globular density at the base of each spoke, although the modeled molecular detail remains tentative at the current resolutions^30,31^. Basket-like protrusions likely arise from extensive coiled-coil domains of *S. cerevisiae* Mlp1/2 and human TPR, which interact with the Pml39 and ZC3HC1 homologs, respectively^122–127^. The remaining nuclear basket components, NUP153, and NUP50, or Nup1, Nup2, and Nup60, are largely unstructured and poorly conserved, but share common elements like FG repeats, linker regions, Ran-binding domains, and ZnF domains^26,128–130^. NUP153 and Nup60 are required for TPR and Mlp1/2 attachment to the NPC, respectively^116,131–133^. Consistent with the recurring linker-scaffold architectural principle, biochemical dissection of Nup60 has shown that it presents binding sites for the CNC component Nup85 and nuclear basket-nups Nup2, Mlp1, and possibly Mlp2^133–135^. Additionally, NUP153, Nup1 and Nup60 harbor amphipathic α-helices thought to anchor the nuclear basket to the nuclear envelope^134,136^.

There is a growing appreciation that the nuclear basket links nuclear processes to the NPC, including localizing highly transcribed chromatin regions to the NPC^137–139^. In *S. cerevisiae*, both single and double nuclear outer ring architectures have been observed, with nuclear baskets present in association with either configuration, but nuclear basket-less NPCs consistently displaying only a single nuclear outer ring^28^. *sc*NPCs with nuclear baskets locate away from nucleoli and have been found to recruit distinct RNA and protein interactomes, as well as Mlp1, Pml39, and Nup60, which are required to retain immature transcripts in the nucleus^28,114,116,140,141^. Mlp1 dissociates from NPCs under heat shock, leading to preferential export of heat shock transcripts, but also suggesting that, while they play a regulatory role, nuclear baskets are not essential for mRNA export^142^. Nuclear basket nups are prominent ubiquitination and SUMOylation targets in the cell’s response to various stresses^143,144^. Interestingly, the deSUMOylating enzyme Ulp1/SENP2 localizes to the nuclear basket, where its activity is implicated in gene de-repression^145–147^.

### Nucleocytoplasmic transport

The primary function of the NPC is regulating the exchange of macromolecules between the nucleus and cytoplasm. Its permeability barrier impedes free diffusion of macromolecules in a size-dependent manner^148–150^, with active translocation relying on transport receptors of the karyopherin (kap) family^151^. Translocation is remarkably fast: NPCs transport ~1,000 macromolecules/second with ~10ms cargo dwell times, suggesting concurrent transport of ~10 cargos^152^.

#### The permeability barrier of the NPC

Intrinsically disordered FG-repeat regions that form the permeability barrier constitute ~40% of the *hs*NPC’s mass^153–158^. Historically, this barrier was thought to limit free diffusion of macromolecules >40kDa^159,160^. More recent studies suggest that the barrier affects the diffusion kinetics of a broader range of molecular sizes^161,162^. Composite NPC structures revealed where FG-repeats emanate from, including the cytoplasmic filaments (NUP358, NUP42, and the CFNC), nuclear basket (NUP153, NUP50, and TPR), and inner ring surrounding the central transport channel (NUP98 and the CNT), where the local FG motif concentration is ~50mM (Fig. 4E)^18,19,24,26–28,155,163^. FG-repeat regions appear to be functionally redundant, as suggested by their non-lethal deletion from 11 *S. cerevisiae* FG-nups, in various combinations^164^. Transport factors and energy input enable transport of cargos orders of magnitude greater than the diffusion cutoff, against concentration gradients.

Purified FG-repeats form hydrogels *in vitro*, excluding proteins in a size-dependent fashion while allowing transport factor penetration at near-physiological rates^163,165–169^. FG hydrogels arise from an amyloid-like β-sheet meshwork of interactions^170,171^. Under specific *in vitro* conditions, short NUP98 FG peptides can even form repetitive fibrils^172–175^. In dilute conditions, FG hydrogels assemble into spherical particles, suggesting a liquid transition state that experiences surface tension. Indeed, millisecond-scale studies revealed FG-LLPS that may recapitulate more closely the *in vivo* permeability barrier^176^.

Phenylalanine residues are critical for FG self-association, as serine substitutions or aliphatic alcohols impair hydrogel formation^166,177^. This is consistent with their lower critical solution temperature (LCST) polymer behavior, whereby higher temperatures promote phase separation driven by the unfavorable entropy of polymer solvation^178,179^. FG nups contain different repeat patterns, such as the GLFG, FxFG, or FG, with cohesiveness determined by FG-repeat pattern, density, peptide length, and the length and composition of the inter-FG spacer sequences^170,180,181^. An artificial 12-mer GLFG peptide showed that selective FG hydrogels can be engineered with minimal components^182^.

FG-repeat cohesiveness determines the stringency of the permeability barrier *in vitro*, consistent with a “selective phase” model in which FG regions form multivalent inter- and intra-molecular interactions to generate a size-selective mesh penetrable by transport factors binding to FG motifs^164,166–170,180,184,185^. Cargo association with transport factors permits proteins that would typically be excluded from partitioning into this barrier to translocate the NPC by locally disrupting FG-FG interactions. A competing “polymer brush” model envisions FG regions preventing free diffusion through polypeptide chains behaving like flexible filaments that resist compaction, resulting in an energy barrier that cargo must overcome, typically offset by favorable interactions with transport factors, in concordance with time-resolved *in situ* atomic force microscopy observations of filamentous objects transiently protruding from NPCs’ central transport channel^186–189^. The two models are not necessarily mutually exclusive and may each describe FG nups in different parts of the NPC.

The NPC’s FG-repeat ‘cloud’ presents >1,000 binding sites for transport factors, extending up to ~100nm from the central transport channel^190–192^. Crystal structures reveal how deep hydrophobic clefts on transport factor surfaces specifically recognize FG phenylalanines, whose conformations are unconstrained due to their adjacency to glycine residues, allowing greater backbone flexibility and enabling phenylalanine side chains to extend into these clefts^156,193–196^. Productive interactions with FGs increase cargo transit rates, as shown by engineering the surface of a GFP protein to bind FG motifs via favorable cation-π and π-π interactions^197^. *In vitro*, transport factors can affect FG brush conformation, and present disaggregase activities preventing FG fibril formation^172,188,198–200^. *In vivo*, site-specific fluorescent labeling studies have shown that FG-repeat regions assume extended conformations in the NPC, consistent with inter-molecular binding with other FG peptides and transport factors, rather than self-association^201^. The crowding effect of transport factors in the permeability barrier has also been proposed to increase transport rates^188,202,203^. The anchoring of distinct FG-repeat types suggests a non-uniform distribution, potentially forming distinct FG spatial domains within the diffusion barrier. Whereas recent studies propose specific transit routes through the central transport channel that might be related to non-uniform FG distributions, their functional relevance remains to be established^192,204–206^. While the general principles of the permeability barrier are conceptually understood, ongoing research aims to reconcile the nuanced roles of different FG-repeat types and competing biophysical models in explaining FG-repeat function *in vivo*.

#### Classical nuclear import pathway

**Figure 5 |:**
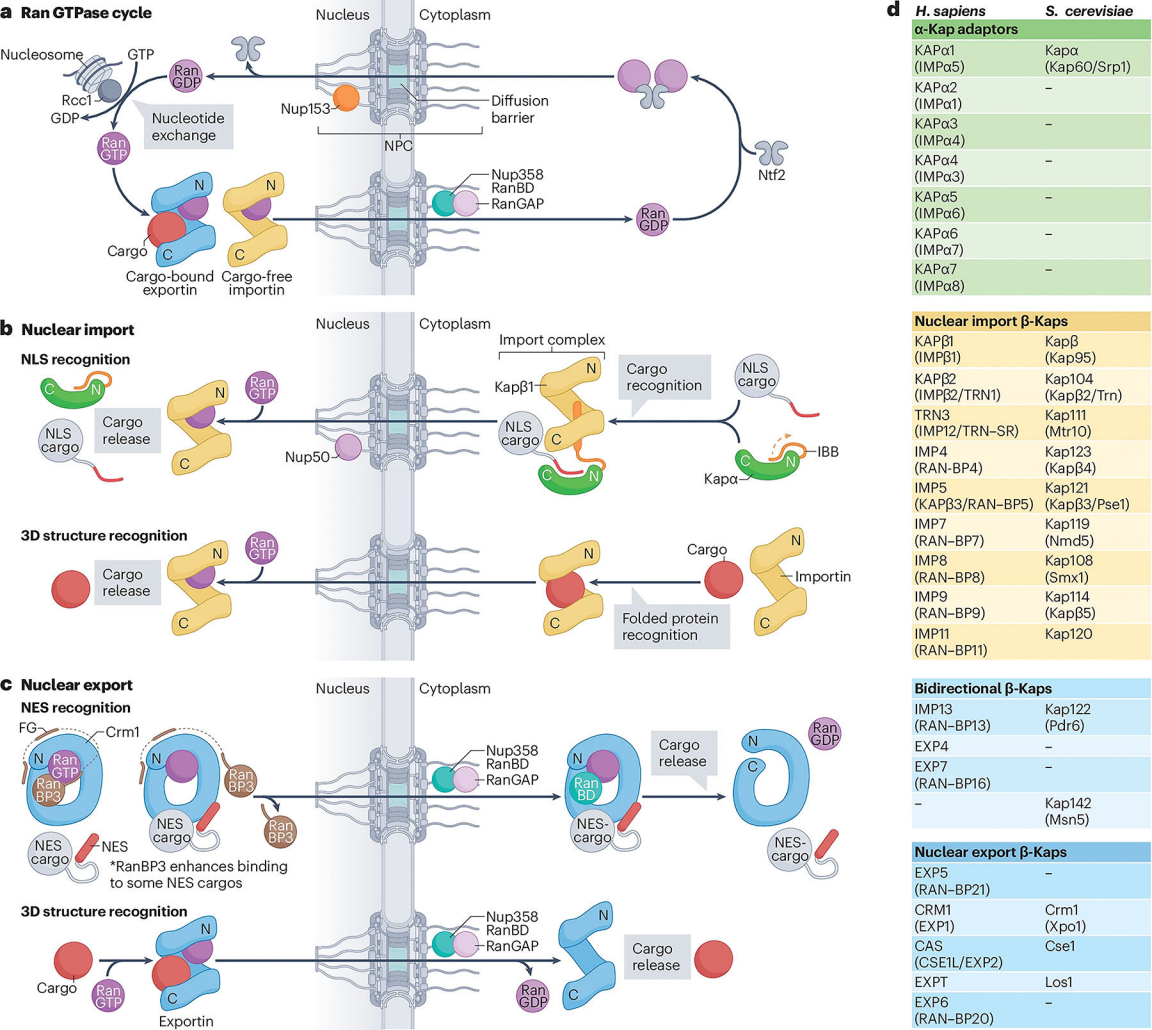
Karyopherin mediated nucleocytoplasmic transport. **a** | Ran GTPase cycle. Nuclear Ran(GTP) is incorporated into β-karyopherin complexes to promote cargo-bound export complex formation, or to trigger cargo release from importins. Once it departs the nucleus by association with export complexes or via passive diffusion, the Nup358-bound GTPase activating protein RanGAP1 stimulates GTP hydrolysis by Ran. This also triggers dissociation of Ran(GDP) from β-karyopherins and the release of export cargo. The *S. cerevisiae* homolog RanGAP1 is cytoplasmic but not associated with the *sc*NPC. Free Ran(GDP) is recognized by cytoplasmic Ntf2 homo-dimers, forming a hetero-tetrameric import complex that crosses the NPC diffusion barrier. At the nuclear basket, Nup153 presents four ZnF domains capable of binding Ran(GDP). The nucleosome-bound Ran guanine exchange factor (GEF), Rcc1, mediates dissociation of GDP from Ran and exchange for GTP, restarting the cycle. **b** | Classical nuclear localization signal (NLS) and 3D cargo recognition. Proteins containing a linear NLS bind to Kapα2, displacing the autoinhibitory importin beta binding domain (IBB), which assumes an α-helical conformation and binds Kapβ1. Transit of this ternary complex across the NPC’s permeability barrier is mediated by interactions between Kapβ1 and FG repeats. Upon nuclear entry, Kapβ1 binds to Ran(GTP) to release the IBB, which alongside Nup50, competes with the NLS to release the cargo. Additionally, many import β-kaps recognize 3D structures such as folded domains in their apo form, with nuclear Ran(GTP) interactions triggering structural rearrangement and cargo release. **c** | Nuclear export signal (NES), and 3D cargo recognition. RanBP3 primes Crm1 for NES-cargo binding. NES-cargo binds the convex face of Crm1, triggering a structural rearrangement that displaces RanBP3. The ternary export complex transits the NPC diffusion barrier by CRM1 interacting with FG repeats. On the cytoplasmic face of the NPC, the NES-cargo•Crm1•Ran(GTP) complex binds to either the RanBD in Nup358 or RanBP1 (not shown). The RanBD/RanBP1 interacts with Ran(GTP), allosterically interfering with NES binding and RanGAP1-stimulated hydrolysis of Ran(GTP) triggers structural rearrangement of CRM1 to release the NES-cargo and Ran(GDP). Additionally, many export β-kaps recognize 3D structures such as folded domains in their Ran(GTP) bound form, with Ran turnover in the cytoplasm triggering structural rearrangement and cargo release. **d** | Overview of transport factors in *H. sapiens* and their respective *S. cerevisiae* orthologs, functionally grouped by α-karyopherin adaptors (green), nuclear import β-karyopherins (yellow), bidirectional β-karyopherins (light blue), and nuclear export β-karyopherins (dark blue). Alternative nomenclature indicated in parentheses. Abbreviations: NPC, nuclear pore complex; NE, nuclear envelope; Ntf2, nuclear transport factor 2; NLS, nuclear localization signal; NES, nuclear export signal; IBB, importin-β binding domain; RanBD, Ran binding domain; RanGAP1, Ran GTPase activating protein 1; RanBP1, Ran binding protein.

The import cycle involving karyopherin-α2 (Kapα2) and karyopherin-β1 (Kapβ1) was the first characterized ‘classical’ nucleocytoplasmic transport pathway^152,207,208^. The histone chaperone nucleoplasmin was used in early nuclear import studies, and its basic, trypsin-cleavable sequence was found to be necessary for nuclear targeting in *X. laevis* oocytes, uncovering a general mechanism for nuclear import^209^. Understanding the underlying transport mechanisms was facilitated by the discovery of constitutively nuclear simian virus 40 (SV40) large tumor antigen (TAg)^210^. TAg mutations abolishing nuclear import were mapped to an unstructured stretch of basic amino acids – the nuclear localization signal (NLS) – which could be fused to cytoplasmic proteins, targeting them to the nucleus^211–213^. Assays that probe selective permeabilization of the plasma membrane led to the identification of factors that target NLS-cargo to the nuclear envelope, to then release it into the nucleus in an energy-dependent manner^214–216^. The nuclear envelope-targeting factors were shown to contain the NLS-binding Kapα2 adapter and Kapβ1^217,218^. The latter belongs to the family of β-karyopherins (β-kaps), which shuttle across the permeability barrier via ultrafast interactions with FG repeats^155,193,219^. The factors stimulating NLS-cargo release from the nuclear envelope were shown to include the GTPase Ran and its dedicated transport factor Ntf2^220,221^. Ran functions as a molecular switch, adopting different conformations depending on whether it is bound to GTP or GDP^222,223^. The spatial segregation of the chromatin-bound Ran guanine nucleotide exchange factor (GEF) Rcc1 in the nucleus, and the Ran GTPase-activating protein (GAP) RanGAP in the cytoplasm, ensures high levels of Ran(GTP) and Ran(GDP) in the nucleus and cytoplasm, respectively (Fig. 5A)^224,225^. In the nucleus, Ran(GTP) induces a conformational change in Kapβ1 that releases an autoinhibitory region of Kapα2, which in turn competes with the NLS to liberate the cargo^226^. Nuclear basket-nups Nup2 and NUP50 harbor Kapα2-binding sites that accelerate cargo release^227–229^. The Kapβ1•RAN(GTP) complex can then cross the NPC’s permeability barrier, to be dismantled by Ran hydrolysing GTP once stimulated by RanGAP in the cytoplasm (Fig. 5B)^225,230^. Antithetically to its import, the released Kapα2 is exported from the nucleus by forming a heterotrimeric complex with the β-kap Cas and Ran(GTP)^231^. This export mechanism was shown to apply to general cargos displaying amphipathic α-helical nuclear export signals (NESs), which in the nucleus form ternary complexes with the β-kap Crm1 and Ran(GTP), and are dismantled by RanGAP’s stimulation of RAN’s GTPase activity in the cytoplasm (Fig. 5C)^232,233^.

#### Transport factor diversity

Approximately a third of human proteins are transported into the nucleus, but only a fraction use classical NLS/NESs^234^. Experimental and bioinformatic approaches have identified 7 α-kaps and 17 β-kaps in humans, as well as 1 α-kap and 14 β-kaps in *S. cerevisiae* (Fig. 5D). Given the number and structural diversity of nuclear cargos, this relatively small number of kaps functions by adopting a broad spectrum of cargo recognition modes ranging from linear NLS/NES peptides, to the use of adapter proteins, to the direct recognition of 3D folds of individual proteins or macromolecular assemblies^152^. This versatility requires kaps to bind many different cargoes with high specificity. High throughput proteomics approaches revealed that kaps have extensive cargo recognition landscapes, as in the case for EXP7 and IMP13, each reported to bind >200 import and export cargos^235,236^. Non-classical cargo-kap binding rules continue to be discovered, as highlighted by the recent findings that circular RNA export is mediated by α-kap exporter Cas^237^, that α-kaps can recognize 3D motifs in WW domains^238^, or that β-kaps moonlight in non-transport functions^239–242^.

#### mRNA export

An outlier to kap-mediated nucleocytoplasmic transport reliant on Ran(GTP) hydrolysis is messenger RNA (mRNA) export^243–246^. Nascent pre-mRNA transcripts are modified with a 5’ 7-methylguanosine cap and then protected by the cap-binding complex (CBC)^247–249^. The pre-mRNA is typically loaded with serine-arginine (SR)-rich proteins, undergoes co-transcriptional splicing which results in the loading of exon-junction complexes (EJCs), and has its 3’ end cleaved and polyadenylated to enable the recruitment of poly-A binding protein 1 (PABPN1) and Nab2^250–254^. This assembly is recognized by Yra1/ALYREF, recruiting the DEAD-box ATPase Sub2/UAP56 and the THO complex to form the transcription-export (TREX) complex^255–260^. Cryo-EM/ET analyses of this mRNP indicate varying factor stoichiometries and a globular yet heterogeneous RNA structure, with Yra1/ALYREF linking RNA-bound EJCs to THO•Sub2/UAP56 complexes at the mRNP surface^261^. To license the mRNP for export, Sub2/UAP56 catalyzes an energy-dependent step, replacing THO•Sub2/UAP56 with transport factors Mex67•Mtr2/TAP•p15 (also known as NXF1•NXT1) through a poorly understood mechanism^262,263^. Mex67•Mtr2/TAP•p15 present the same protein fold as Ran’s importer Ntf2 and interact with FG repeats to facilitate transit through the permeability barrier^194,264–266^.

Export-licensed mRNPs are targeted to the nuclear basket for final maturation and export. Nab2 interacts with the nuclear basket-nup Mlp1^118,141,267,268^. Because the nuclear basket remains biochemically and structurally understudied, other nuclear basket-nups may also turn out to play a role in mRNA export. As they exit through the NPC’s central transport channel, highly transcribed Balbiani ring mRNPs in the *Chironomus* gnat salivary gland have been observed^269,270^. At the cytoplasmic face, the DEAD-box helicase Dbp5/DDX19, and its activator Gle1/GLE1 that binds inositol hexakisphosphate (IP_6_) in fungi but not humans, hydrolyze ATP to displace the Mex67•Mtr2/TAP•p15 and Nab2 factors and irreversibly release mRNA into the cytoplasm^41,95,96,101,271^. The CFNC’s coiled coil hub domain suspends DDX19 over the central transport channel in a crane-like fashion (Fig. 4B), placing NUP214’s N-terminal domain, which modulates DDX19 ATPase activity, in close proximity^24,26^. By contrast, the *hs*NPC composite structure indicates, despite map resolutions that limit the accurate modeling of the GLE1•NUP42 orientation, that GLE1•NUP42 binds below the cytoplasmic outer ring, at bridging NUP155 sites^24,26,41^. This spatial separation suggests that unbinding and repositioning of either GLE1•NUP42 or DDX19 would be required for their direct interaction, which has been otherwise characterized in isolation^41,101,272,273^. Curiously, *ct*Gle1•Nup42 interacts directly with the *ct*CNC in an IP_6_-dependent manner^26^. Whether the spatial arrangement of DDX19 and its ATPase activity modulators reflects a regulated molecular mechanism involving repositioning or cycling of these factors during mRNA remodeling remains an intriguing open question. Dbp5/DDX19 activity is also stimulated by RNA, reaching ~1s^−1^ ATP turnover rates *in vitro*, which is significantly slower than the 20–200ms dwell times of mRNA at the NPC measured *in vivo*^41,272,274–276^. Such inconsistency suggests that a complete understanding of how Dbp5/DDX19 catalysis is coordinated at the NPC has yet to be achieved.

The role of the additional RNA-binding interfaces in the NPC is also poorly understood^26,41,93,99,100^. Prominently, Gle2/RAE1, recruited to a motif nested within the Nup116/NUP98 FG-repeat region, remains a protein of unknown function despite being targeted by diverse viral virulence factors which disrupt mRNA export^91,100,277–280^. Curiously, cryo-EM structures of pre-60S ribosomal particles trapped in purified *sc*NPCs reveal that components of the mRNA export machinery, including Mex67•Mtr2 and Gle2, are bound during transit^281^. Ribosome export was previously shown to require Ran-dependent β-kaps^282–284^, suggesting that some cargos might use both nuclear export pathways.

#### Dilation of the central transport channel

*In situ* imaging reveals an ~650Å-wide central transport channel in sc/*hs*NPCs, which constricts to ~450Å and ~425Å when imaged from *hs*NPCs in isolated nuclear envelopes or detergent-extracted isolated *sc*NPCs, respectively^22,24,27,28^. The linker of the nucleus and cytoskeleton (LINC) complex tethers the nucleus and the cytoskeleton, transferring mechanical forces, both constitutively generated and experienced by the cell, onto the nuclear envelope^285–288^. It is thus possible that membrane tension in the nuclear envelope pulls on the NPC’s inner ring, dilating the central transport channel. Experiments in *S. pombe* confirmed NPC dilation can be altered through hypoosmotic shock or energy depletion^23^. Under extreme dilation, NPC spokes can form “cracks” that allow expansion beyond the limits of the linker-scaffold architecture, a feature probably important to accommodate unusually large macromolecules, such as intact HIV capsids transiting through the central channel^33^.

Dilation of the central transport channel suggests that the NPC integrates mechanical forces experienced by the cell and nucleus, resulting in altered nucleocytoplasmic transport. Cells under mechanical stress, such as during cell migration or differentiation, might adapt their nucleocytoplasmic transport to maintain homeostasis, a phenomenon that might also be exploited by malignancies and pathogens. Steady-state localization of cargos with specific physical properties can be modulated by applying force to the nucleus or culturing cells on substrates with defined rigidity or adhesion geometry^289–291^. However, how varying central transport channel diameters affects the FG-repeat permeability barrier to modulate nucleocytoplasmic transport remains unclear.

### NPC assembly, disassembly and surveillance

**Figure 6 |:**
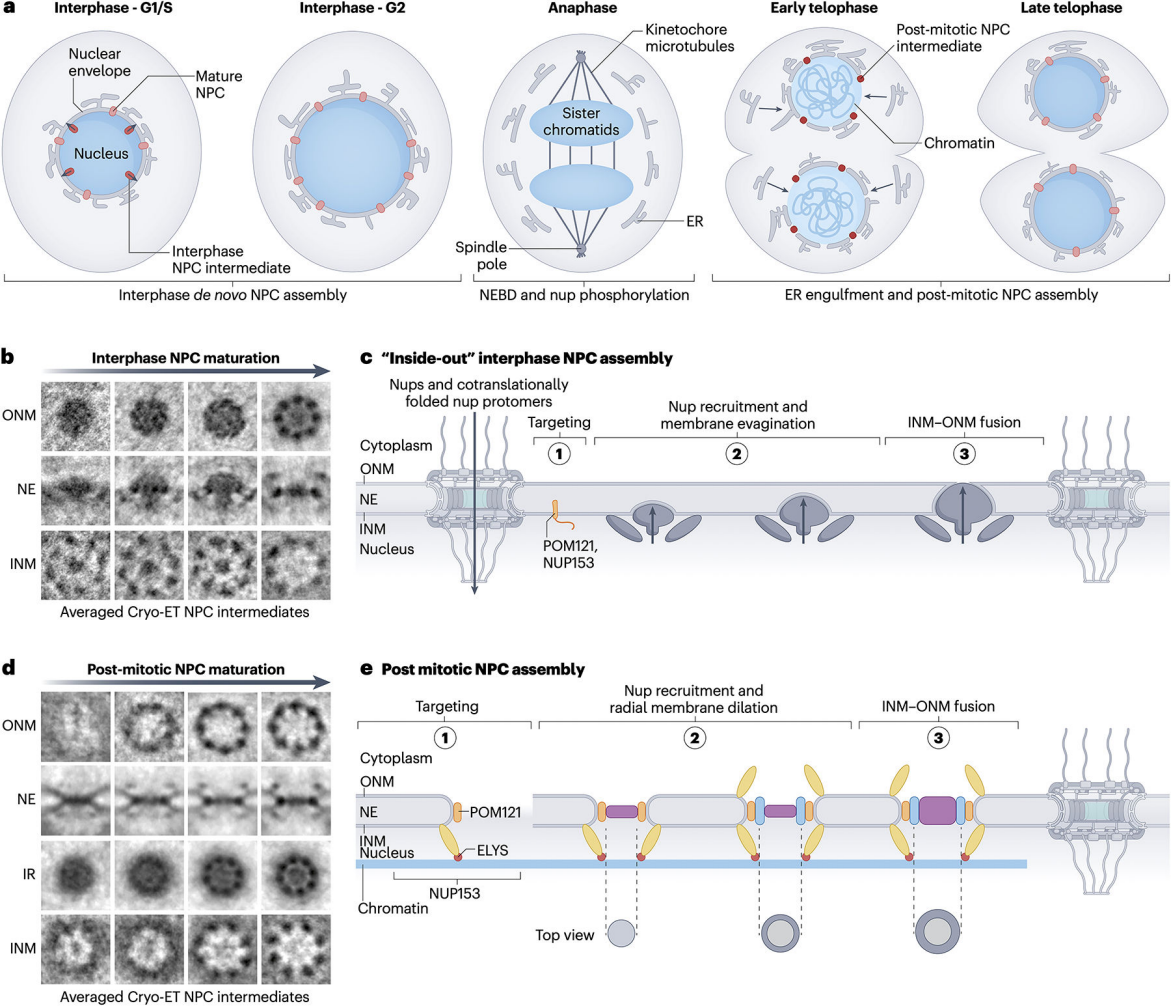
NPC assembly mechanisms. **a** | Cartoon schematic of NPC biogenesis at different stages of the cell cycle. **b** | Averaged cryo-ET recostruction slices of interphase NPC assembly intermediates and mature NPCs. Figure adapted from Otsuka *et al*^310^. **c** |Cartoon schematic of interphase *de novo* NPC assembly. During interphase, the assembly of new NPCs requires nuclear import of nups and pre-assembled nup complexes. Upon nuclear localization, nups are targeted to the INM via a poorly understood mechanism involving POM121 and NUP153. NPC intermediates assigned to early assembly steps include 8-fold symmetric nuclear ring-like structure and a mushroom-shaped inside-out exvagination of the INM. Nup incorporation correlates with the growth of the INM exvagination, with concomitant ONM deformation. Fusion of the INM-ONM is driven by ESCRT-III and VPS4 with the involvement of Brl1/6 and Aqi12 in *S. cerevisiae*, and Torsin-A in *H. sapiens*. NPC assembly intermediates classified to late stages exhibit an 8-fold symmetric cytoplasmic ring, and are proposed to recruit the remaining cytoplasmic filament nups prior to reaching maturity. **d** | Averaged cryo-ET recostruction slices of post-mitotic NPC assembly. Figure adapted from Otsuka *et al*^345^. **e** | Cartoon schematic of post mitotic NPC assembly, CNC (yellow), ELYS (red), POMs (brown), IRC (blue), and the central transport channel (purple). Upon exit from mitosis, NPC assembly proceeds concurrently with NE reformation. Nups are recruited in a stepwise sequential as the ER engulfs naked chromatin. This process is initiated by the chromatin-associated nuclear outer ring nup ELYS. At the end of mitosis, LEM2 engages ESCRT-III and CHMP7 to seal the membrane, whereas SPASTIN severs the connected microtubules. Abbreviations: NPC, nuclear pore complex; NE, nuclear envelope; ONM, outer nuclear membrane; INM, inner nuclear membrane; cryo-ET, cryo–electron tomography.

Cells surveil and maintain NPC numbers, composition, and functionality at steady state, and must double their NPC count before cell division^292,293^. Rapid NPC reassembly after open mitosis (whereby the nuclear envelope disassembles entirely at the start of mitosis and reforms after chromosome segregation) and *de novo* NPC biogenesis during interphase occur via distinct assembly pathways (Fig. 6A)^294–297^. A third pathway involves pre-assembly of NPC precursors (AL-NPCs) in endoplasmic reticulum membrane stacks called *annulate lamellae*^8^.

#### Nup synthesis and protomer assembly

Nup overexpression can alter endoplasmic reticulum and nuclear envelope morphology and cause toxic kap-sequestering cytoplasmic foci^42,135,298–302^. In *S. cerevisiae*, nup mRNA levels are kept constant by general regulatory factors Abf1 and Reb1, and the mRNA-binding translational repressor Hek2^301,303–305^. In humans, however, select nup mRNA levels vary across tissues^112^.

There are no known dedicated NPC assembly chaperones. Co-translational binding between inner ring nups promotes protomeric subcomplex assembly before *sc*NPC incorporation^306^. Pre-assembly expedites and hierarchizes nup incorporation, potentially directing assembly of paralogous nups with divergent roles, such as the Nup100/116/145N linkers, which share some but not all binding partners^27,306^. Consistent with the observation that import kaps surveil nascent proteins at the ribosome, co-translational kap recognition of Nup1 and Nup2 facilitates their nuclear import and nuclear basket localization^302,306^. Pulse-chase experiments place nup subcomplex formation on the minute timescale, whereas NPC incorporation is an order of magnitude slower^307,308^.

#### De novo NPC assembly during interphase

Interphase assembly is considered the sole NPC biogenesis pathway in terminally differentiated cells and organisms with closed mitosis (whereby the nuclear envelope remains intact throughout the process of chromosome segregation)^309^. Microscopy reveals an “inside-out” mechanism where nascent NPCs extrude through the nuclear envelope by evaginating the INM and fusing it with the ONM (Fig. 6B-C)^310^. Tracing GFP-tagged nups in HeLa cells established an ~hour-long timeline of NPC incorporation, starting with pioneering POM121 and nuclear basket-nups, followed by outer ring and inner ring components, and ending with NUP358^311^. A sequential inside-out model for interphase *sc*NPC assembly in closed mitosis is supported by deletions of either Nup116 or Nup170/157 accumulating aberrant *sc*NPC precursors at the INM^312,313^. In both cases, the inside-out mechanism requires import of at least some nups via pre-existing NPCs. Fragmented evidence, including that disruption of Ran and Kapβ affects *sc*NPC assembly^314,315^, and that NUP153 and POM121 respectively recruit CNC and IRC protomers to the INM in human cells^137,316,317^, supports the model that nups are imported to the nucleus via pre-existing NPCs then are assembled into new NPCs, starting at the INM.

In *S. cerevisiae*, the transmembrane proteins Brl1, Brl6 and Apq12 are implicated in INM-ONM fusion^318,319^. In human cells, the AAA+ ATPase Torsin-A and LAP1/LULL1 cofactors have a similar role^320,321^. Torsin-A mutations causing “Ω”-shaped NE herniations with nup accumulation at the base are associated with *DY*T-*TOR1A* dystonia^322–325^. These herniations contain DNAJB6 and other HSP70-HSP40 chaperones, which interact with FG-nups and can prevent their aggregation^326,327^. Despite these insights into *de novo* NPC assembly timelines, the underlying molecular mechanisms remain unclear. Trapping assembly intermediates with anti-nup nanobodies may provide a way to dissect them^328^.

#### Mitotic NPC disassembly

NPCs are remarkably robust, with dwell times of inner ring and outer ring scaffold nups persisting entire cell cycles, and some nups’ NPC residency lasting the lifetime of terminally differentiated cells^191,309,329,330^. Open mitosis requires active NPC disassembly followed by reassembly upon nuclear envelope reformation. NPC disassembly begins with nuclear envelope breakdown during prophase, initiated by phosphorylation of NPC and nuclear lamina components, though these may be temporally overlapping but separate pathways^331,332^. Triggered by cell-cycle dependent kinases, NPC disassembly occurs within minutes, starting with NUP98 dissociation^333^.

During prophase, CDK1, PLK1, and the NIMA-family kinases extensively phosphorylate the scaffold-binding regions of linkers NUP53 and NUP98^71-74^. Mitotic phosphorylation of unstructured regions in CNC components has also been observed, though the responsible kinase(s) are unknown^334^. Released subcomplexes resemble co-translationally assembled NPC protomers, with the CNC and NUP93•CNT complexes remaining intact, and a subset of CNCs remaining associated with the endoplasmic reticulum throughout mitosis^334–336^.

#### Post-mitotic NPC reassembly

At the end of open mitosis, the nuclear envelope reforms as endoplasmic reticulum membranes envelop chromatin^337^. Reversing disassembly, nuclear envelope constituents are dephosphorylated before incorporation, driven in *C. elegans* by protein phosphatase 1 (PP1), which is recruited to an unstructured C-terminal region of ELYS that is conserved across metazoans^295,338,339^. Post-mitotic NPC assembly appears stepwise, initiated by keystone interactions between CNCs and the chromatin-bound ELYS (Fig. 6D-E)^333,340–345^. Unlike interphase assembly, this process uses stockpiled nups, with NPCs forming even under translation inhibited conditions^292^. Besides ELYS, NDC1 and POM121 play important roles in post-mitotic reassembly by respectively recruiting the linker NUP53 and the scaffolds NUP160 and NUP155^76,84^. Tracing GFP-tagged nups post-mitosis further suggests that interphase and post-mitotic NPC assembly differ in nup incorporation order^311^.

Coordinating nuclear envelope reformation and NPC reassembly is crucial for nuclear integrity and function. Knockdown of CNC components NUP133 and NUP107 lead to NPC-devoid nuclear envelopes, a phenotype not rescued by restoring these nups after late-anaphase nuclear envelope sealing^346^. The endosomal sorting complex responsible for transport (ESCRT)-III, the AAA+ ATPase VPS4, CHMP7, and SPASTIN are recruited to microtubule engulfment nuclear envelope sites^347,348^. Upon mitotic exit, SPASTIN severs microtubules, while ESCRT-III seals the membrane^347,348^. A factor in the INM, LEM2, targets ESCRT-III to the nascent nuclear envelope and activates CHMP7^349^. Premature membrane fusion is prevented by CDK1 phosphorylation of LEM2 throughout mitosis^350^.

A Ran(GTP) gradient, established by the Ran GEF Rcc1 and RanGAP, is crucial for nuclear envelope reformation, as shown by reconstitution in *X. laevis* oocyte extracts^351,352^. β-kaps negatively regulate NPC assembly, chaperoning nups until released by Ran(GTP), even though nup-kap interactions have not yet been mapped systematically^353–355^. Known examples include Ran(GTP)-dependent incorporation of Kapβ1-chaperoned CNCs into the *xl*NPC^356^, Elys-Kapβ1/β2 interactions competitive with Elys’ chromatin association in *X. laevis* oocytes^356,357^, and Kap121/Kapα binding to Nup53/59’s scaffold-binding regions in *S. cerevisiae*^36,358^. Feedforward loops likely coordinate assembly, as with *X. laevis* nuclear basket-nup Nup50 activating Rcc1 to spatially coordiate Ran(GTP) gradient establishment at NPC assembly sites^359^.

#### AL-NPC assembly

Pre-assembly of AL-NPCs in *annulate lamellae* membrane sheets allows their *en bloc* insertion into the nuclear envelope via poorly understood mechanisms^360^. *Annulate lamellae*, found in oocytes, spermatocytes, some somatic and cancer cells, are a hallmark of rapid cell division that would outpace *de novo* nuclear envelope synthesis and NPC assembly^361^. AL-NPC incorporation into the nuclear envelope has been primarily studied in *D. melanogaster* blastoderm embryos, where AL-NPCs are devoid of CNTs lining their central transport channel and of most cytoplasmic filament- and nuclear basket-nups, but notably have Nup358 symmetrically attached to both outer rings^360^. Maternal nups, abundant in the *D. melanogaster* ooplasm^362^, can presumably convert AL-NPCs into fully functional, asymmetric NPCs. By contrast, AL-NPCs from *X. laevis* stage VI oocytes already harbor the CNT/CFNC component Nup62^363^, suggesting AL-NPC composition may vary by species or developmental stage.

AL-NPC assembly in *D. melanogaster* oogenesis is mediated by kaps, Ran, and Nup358, as well as condensed nup granules supplied by both oocytes and nurse cells connected to oocytes via cytoplasmic bridges termed ring canals^364^. Granule formation and transport on microtubules may localize and concentrate nups to AL-NPC assembly sites in the absence of chromatin, which typically anchors post-mitotic NPC reassembly. Nup358’s prominent role in AL-NPC assembly contrasts with NUP358’s late addition to nascent *hs*NPCs in interphase and post-mitotic assembly^311,364^.

## Conclusions and perspectives

Recent advances in genetic, biochemical and structural studies of nups, combined with cryo-ET reconstructions of intact NPCs, have advanced our understanding of NPC architecture in fungi and metazoans. These developments have enabled comparative analyses across species, shedding light on conserved and divergent features of NPCs. NPCs exhibit a modular architecture, integrating rigid scaffolds with flexible linker nups to allow for dilation in response to nuclear envelope tension. While the symmetric core of the NPC is largely conserved across species, notable variations are observed in the cytoplasmic filaments, nuclear basket, and POM regions, reflecting species-specific adaptations. Although many aspects of nucleocytoplasmic transport are conceptually well understood, several questions remain unresolved. These include uncovering the molecular basis of mRNA export, elucidating how transport factors achieve remarkable specificity in binding diverse cargos to regulate their nucleocytoplasmic distribution, determining how disease-associated mutations in nups alter NPC function, affect nucleocytoplasmic transport rates and selectivity, and ultimately contribute to disease pathology. Moreover, despite some progress, the mechanisms governing NPC assembly, surveillance, and repair remain poorly understood. Near-atomic composite structures of NPCs provide essential blueprints for mechanistic studies that will enable to study such mechanisms and adress open questions (Box 1).

## Box 1 | Open questions and future directions in the study of nucleocytoplasmic transport

We have selected open questions that we believe will guide future research to understand NPC function and mechanisms, and their relevance to disease:

- What is the structure and nup interactome of the NPC’s POM and nuclear basket regions?
- Given the longevity of certain nups and the complexity of the NPC’s architecture, what is the impact of defective NPCs on ageing and ageing-related diseases?
- What is the molecular mechanism that enables mRNA export at physiological rates?
- How is the remarkable specificity of transport factors for a broad spectrum of cargos structurally encoded?
- What is the role of post-translational modifications in the NPC’s architecture, assembly, and disassembly?
- What is the compositional heterogeneity of NPCs, and what are the implications for NPC function? Conversely, do “specialized” NPCs with distinct compositional makeups that are related to their physiological function exist?
- How is NPC assembly regulated in different contexts? To what extent is assembly hierarchical and regulated by assembly chaperones, and which events during assembly are spontaneous equilibrium phenomena?
- How are NPCs surveilled, and by what factors? How are assembly defects corrected or defective components of the NPC exchanged? What processes lead to whole-NPC autophagy and how are they regulated?
- Can NPC assembly, dynamics, composition, and function be targeted with drugs to therapeutically modulate nucleocytoplasmic transport in diseases linked to NPC dysfunction, such as cancer, viral infections, or neurodegenerative disorders?
